# Supplementary material for: Essential role of submandibular lymph node dendritic cells in protective sublingual immunotherapy against murine allergy
Source: Commun Biol. 2020 Dec 7;3:742. doi: 10.1038/s42003-020-01466-3 (PMC7721894; doi:10.1038/s42003-020-01466-3)
Supplement: Supplementary file 1 — Supplementary Information [file 42003_2020_1466_MOESM1_ESM.pdf]

Supplementary Information

Essential role of submandibular lymph node dendritic cells in protective sublingual immunotherapy against murine allergy

Noriaki Miyanaga, Hideaki Takagi, Tomofumi Uto, Tomohiro Fukaya, Junta Nasu, Takehito Fukui, Yotaro Nishikawa, Tim Sparwasser, Narantsog Choijookhuu, Yoshitaka Hishikawa, Takeshi Nakamura, Tetsuya Tono & Katsuaki Sato

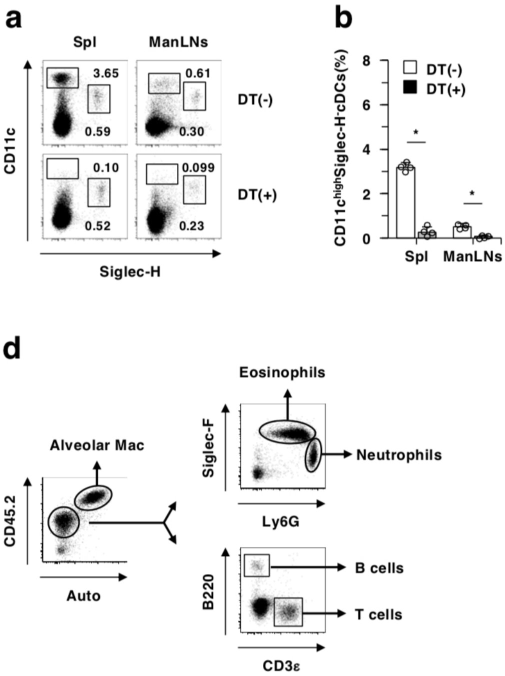

Supplementary Figure 1 Elimination of cDCs in ManLNs in cDC-ablated mice. CD11c-DTR/EGFP mice were treated with PBS or DT, and various tissues were obtained on the next day after DT treatment. Cell surface expression profile (**a**) and proportion (**b,c**) of CD11c^{high}Siglec-H^{-} cDCs (**a,b**) and CD11c^{int}Siglec-H^{+} pDCs (**a,c**) among leukocytes in Spl and ManLNs at the next day after DT treatment. (**d**) BALF cells were analyzed in the indicated sequential gates for CD45.2 and autofluorescence (Auto) to exclude alveolar macrophages. For the detection of other leukocytes, CD45^{+}Auto^{-} cells were further analyzed to identify Ly6G^{high}Siglec-F^{mid} neutrophils, Ly6G^{mid}Siglec-F^{high} eosinophils, B220^{+} B cells, and CD3ε^{+} T cells. Data are obtained from five individual samples in a single experiment. *$P < .05$ compared with CD11c-DTR/EGFP mice that had been treated with PBS indicated as DT(-). All data are representative of at least three independent experiments.

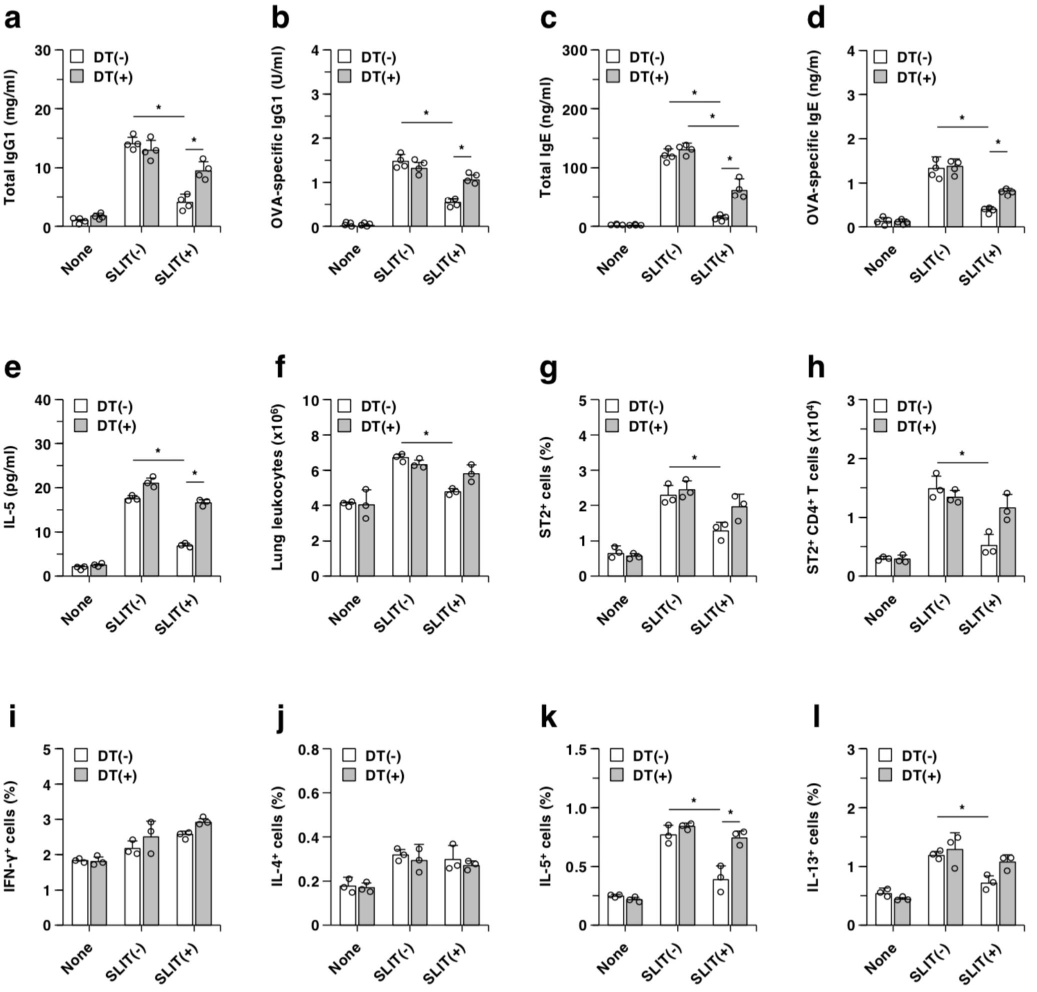

Supplementary Figure 2 Deficiency of cDCs abrogates the inhibitory effect of SLIT on Ag-specific T_H2-responses. CD11c-DTR/EGFP mice that had been treated with PBS or DT were sublingually administrated with PBS or OVA protein, and then systemically immunized with or without OVA protein at 7 and 14 days after SLIT. Subsequently, mice were i.n. sensitized with or without OVA protein at 10, 11, and 12 days after the last immunization, and serum and lung tissue were obtained at 13 days after the last sensitization. **(a-d)** Serum production of total IgG_1 (**a**), OVA-specific-IgG_1 (**b**), total IgE (**c**), and OVA-specific IgE (**d**). **(e)** Serum production of IL-5. **(f-h)** Absolute cell numbers of leukocytes (**f**), proportion of $ST2^+CD44^+$ cells among $CD4^+$ T cells (**g**), and absolute cell number of $CD4^+ST2^+CD44^+$ T cells (**h**) in lung. (**i-l**) Proportion of the intracellular cytokine expression profile of lung $CD4^+$ T cells. $*P < .05$ compared with CD11c-DTR/EGFP mice that had been treated with PBS indicated as DT(-) or among groups. All data are representative of at least 3 independent experiments.

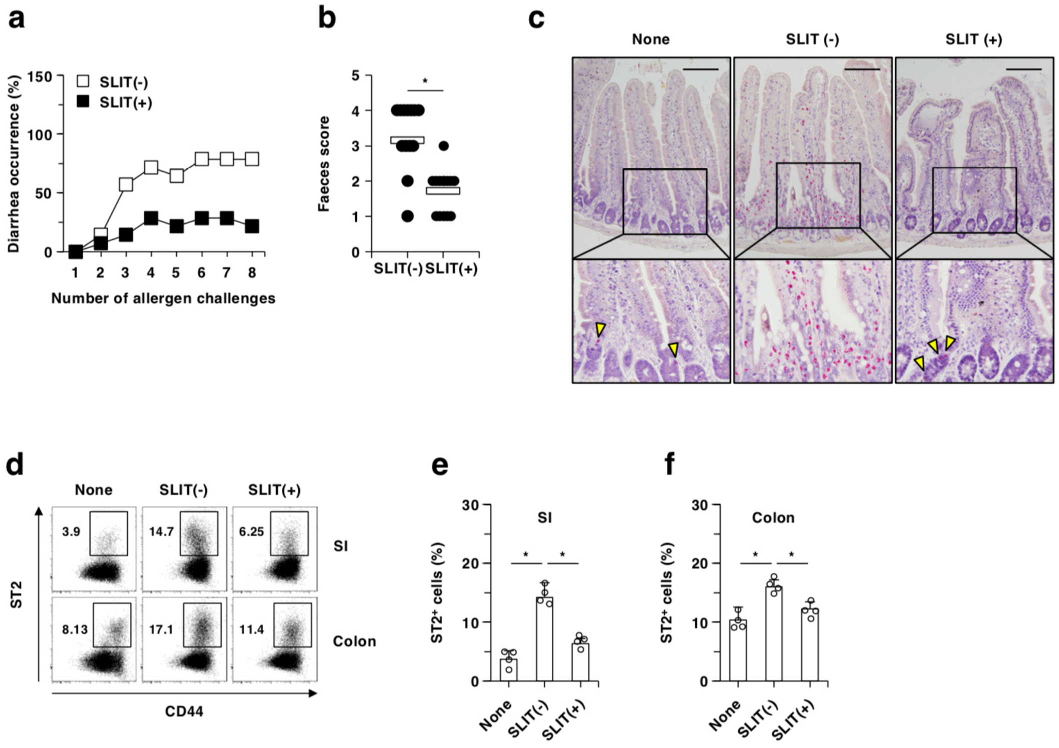

Supplementary Figure 3 SLIT inhibits the development of T_H2-mediated food allergy. WT mice were sublingually administrated with PBS or OVA protein, and then systemically immunized with OVA protein at 7 and 21 days after SLIT. From 14 days after the last immunization, mice were i.g. challenged with OVA protein every 2 days at total of eight times. (**a,b**) Diarrhea occurrence (**a**) and fecal score (**b**). (**c**) Sections obtained from SI were stained for Chloro-acetate esterase to detect intestinal mast cells (red). Bars = 100 μm. Arrow indicates the low frequency of mast cells in the sections of normal mice (None) and immunized mice received SLIT indicated as SLIT(+). (**d-f**) Cell surface expression profile (**d**) and proportion (**e,f**) of $ST2^+CD44^+$ cells among $CD4^+$ T cells in small intestine (SI) (**d,e**) and colon (**d,f**). Data are obtained from five individual samples in a single experiment. *P < .05 compared with untreated immunized mice indicated as SLIT(-) (**b**), or normal mice (None) (**e,f**), or among groups(**e,f**). All data are representative of at least three independent experiments.

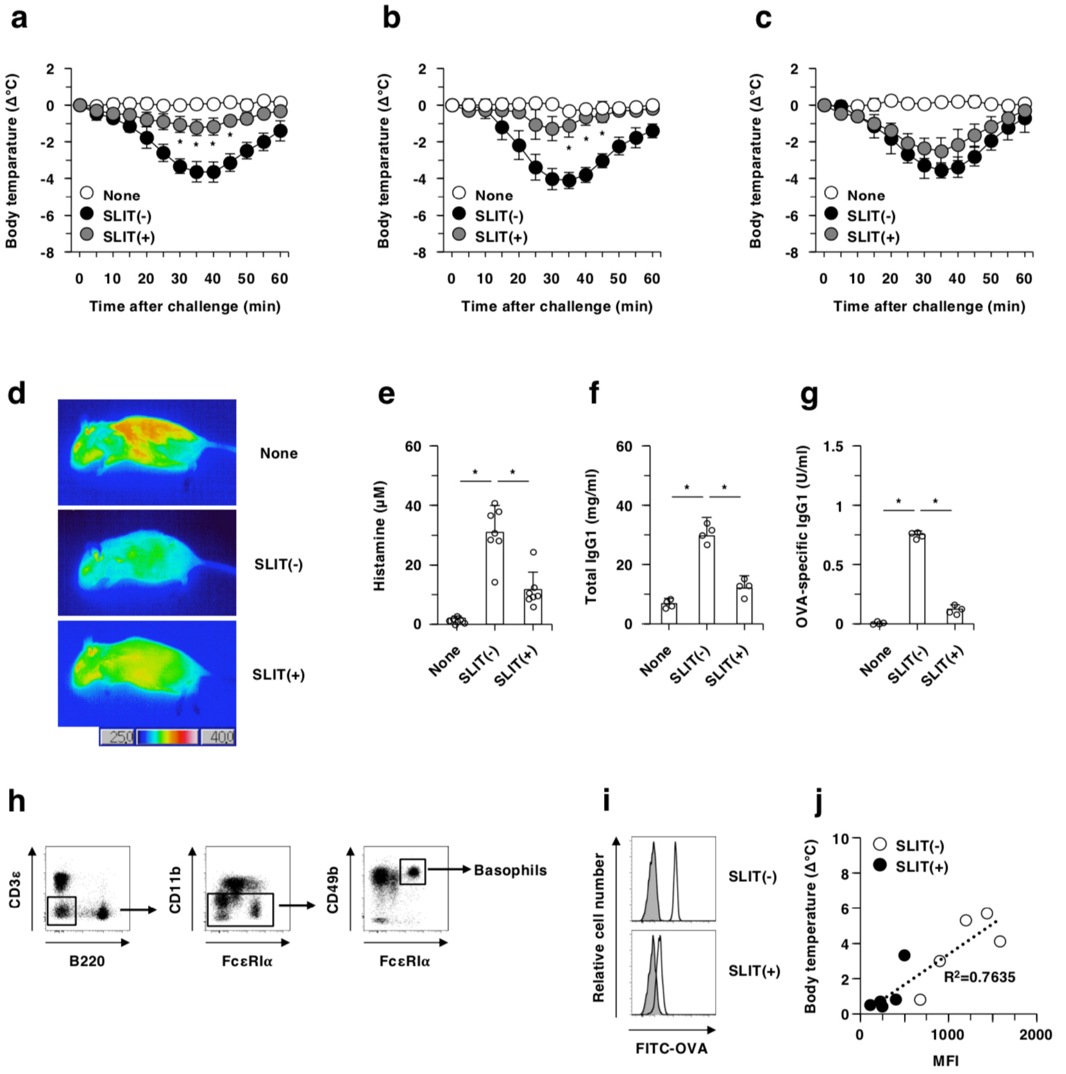

Supplementary Figure 4 SLIT suppresses the development of T_H2-mediated systemic anaphylaxis. WT mice (**a,d-j**) and CD11c-DTR/EGFP mice that had been treated with PBS (**b**) or DT (**c**) were sublingually administrated with PBS or OVA protein and then systemically immunized with OVA protein at 7 and 14 days after SLIT. Subsequently, mice were i.p. challenged with OVA protein 10 days after the last immunization. (**a-c**) Rectal temperature after sensitization. (**d**) Image of whole body temperature at 35 min after sensitization. (**e-g**) Serum level of histamine (**e**), total IgG_1 (**f**), and OVA-specific IgG_1 (**g**). (**h-j**) PBMCs were cultured with FITC-OVA. Cell surface expression profile of $CD3\varepsilon^- B220^- CD11b^- Fc\varepsilon RI\alpha^+ CD49b^+$ basophils (**h**) and their specific binding of FITC-OVA (**i**). (**j**) Correlation diagram of MFI and decline in body temperature (Δ°C). R^2 represents the coefficient of determination. Data are obtained from 5 individual samples in a single experiment. *$P < .05$ compared with untreated immunized mice indicated as SLIT(-) or normal mice (None). All data are representative of at least three independent experiments.

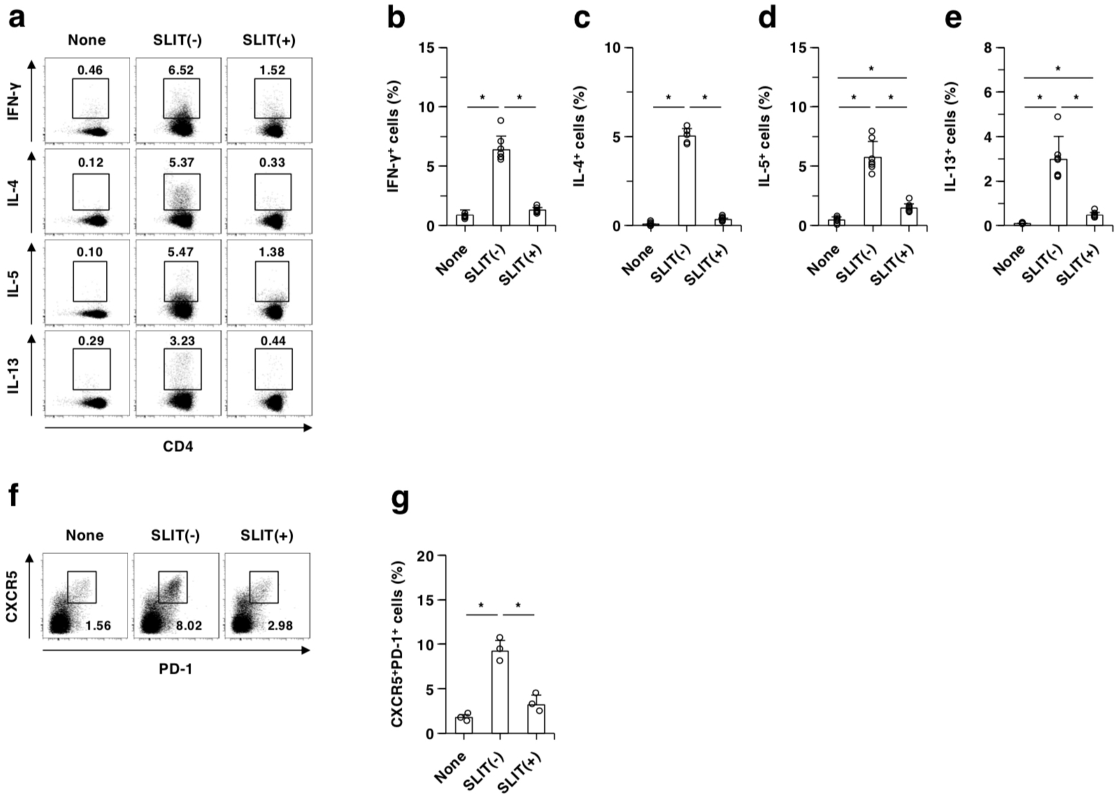

Supplementary Figure 5 SLIT inhibits T_H2-mediated allergic immune responses in mediastinal LNs of asthmatic mice. WT mice were immunized as described in Figure 1 for the induction of allergic airway inflammation. Intracellular cytokine expression profile (**a**) and proportion (**b-e**) of $CD4^+$ T cells in mediastinal LNs. (**f,g**) Cell surface expression profile (**f**) and proportion (**g**) of $CXCR5^+PD\text{-}1^+$ T_{FH} cells among splenocytes. Data are obtained from five individual samples in a single experiment. *$P < .05$ compared with normal mice (None) or among groups. All data are representative of at least three independent experiments.

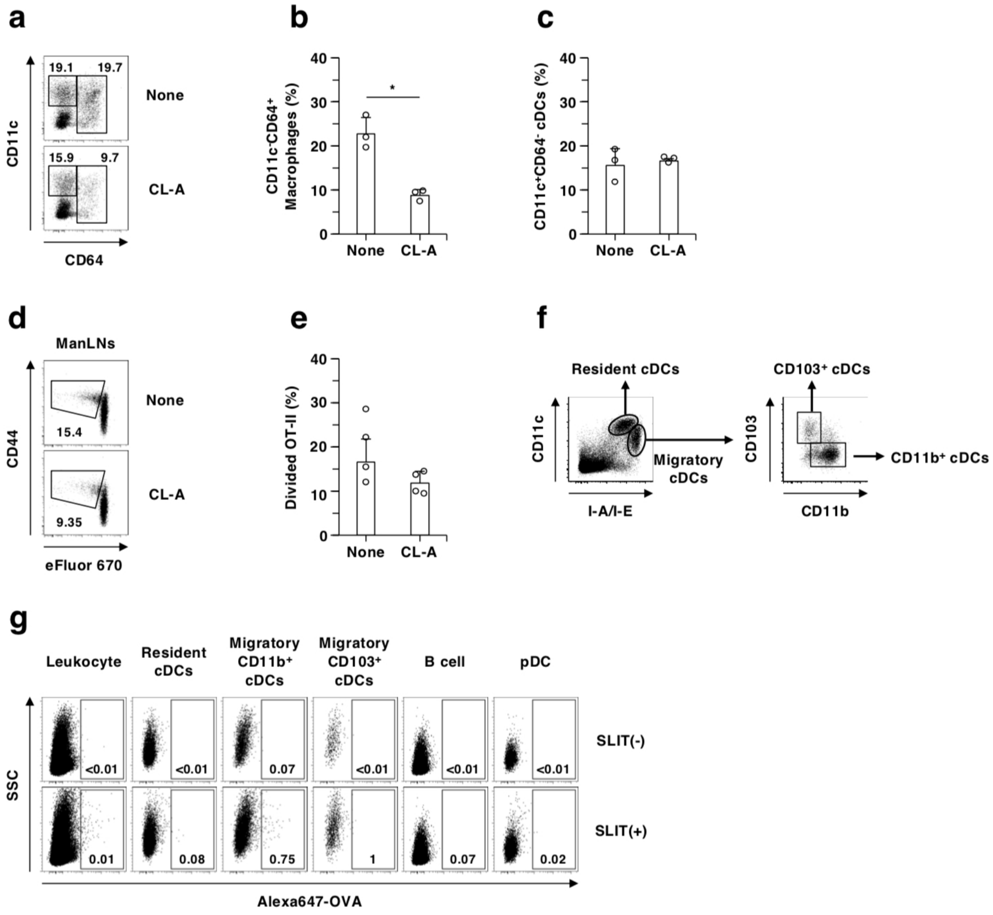

Supplementary Figure 6 Migratory I-A/I-E^{hi}CD11c^{med}CD11b^{+} cDCs retains the sublingual application of Ag. (**a-c**) WT mice were treated with or without CL-A for two consecutive days, and oral tissue were obtained at 24 hours after the final treatment. Cell surface expression profile (**a**) and proportion (**b,c**) of CD11c^{-}CD64^{+} macrophages (**a,b**) and CD11c^{+}CD64^{-} cDCs (**a,c**) among CD45^{+} cells. (**d,e**) WT mice that had been treated with or without CL-A were adoptively transferred with eFluor ™ 670-labelled CD45.1^{+}OT-II CD4^{+} T cells, and then mice were sublingually administered with OVA protein. Cell dividing profile (**d**) and proportion (**e**) of CD45.1^{+}OT-II CD4^{+} T cells in ManLNs at 3 days after the administration. (**f**) Cell surface expression profile of migratory I-A/I-E^{hi}CD11c^{med} CD11b^{+} cDCs, migratory I-A/I-E^{hi}CD11c^{med}CD103^{+} cDCs, and resident I-A/I-E^{med}CD11c^{hi} cDCs in ManLNs. (**g**) WT mice were sublingually administrated with AlexaFluor® 647-labelled OVA protein. Cell surface expression profile of AlexaFluor™ 647-labelled OVA protein-binding cells among leukocytes in ManLNs at 18 hours after the administration. Data are obtained from five individual samples in a single experiment. **P* < .05 compared with untreated mice (None). All data are representative of at least three independent experiments.

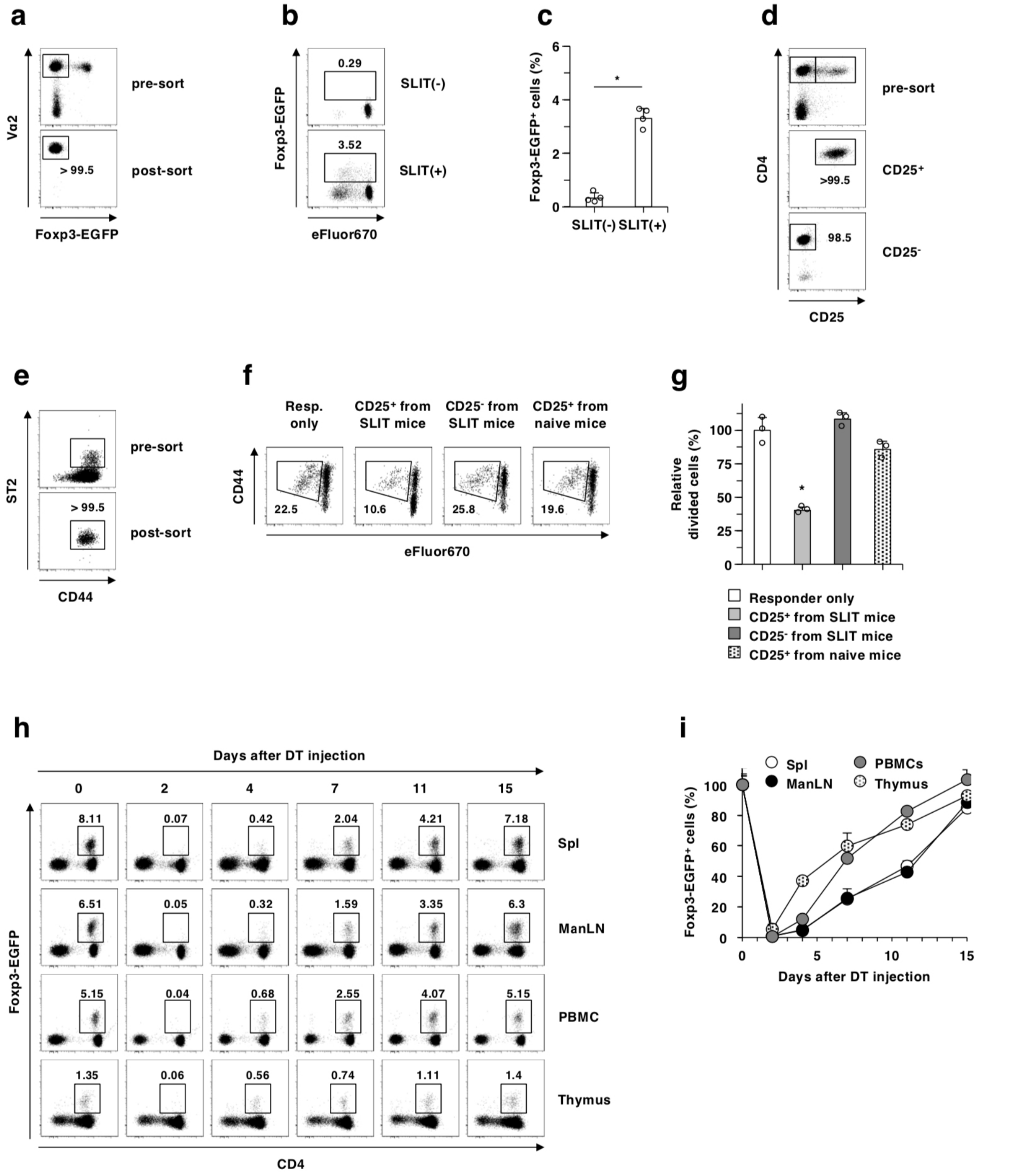

Supplementary Figure 7 CD4$^+$CD25$^+$ T cells suppress the systemic anaphylaxis. (**a**) Cell surface expression profile of CD45.1$^+$Vα2$^+$CD4$^+$*Foxp3*$^{\text{EGFP-}}$ T cells （CD45.1$^+$OT-II CD4$^+$*Foxp3*$^{\text{EGFP-}}$ T cells） isolated from CD45.1$^+$*Foxp3*^{EGFP}CD4$^+$ T cells (CD45.1$^+$OT-II CD4$^+$ T cells). (**b,c**) eFluor™ 670-labelled KJ1-26$^+$CD4$^+$*Foxp3*$^{\text{EGFP-}}$ T cells were cultured with ManLNs cDCs obtained from mice that had been treated with or without sublingual application with OVA protein under pT$_{\text{reg}}$-polarized culture conditions for 5 days in the absence of OVAp. Cell dividing profile **(b)** and proportion **(c)** of KJ1-26$^+$CD4$^+$*Foxp3*$^{\text{EGFP+}}$ T cells. (**d**) Cell surface expression profile of CD4$^+$CD25$^+$ T cells and CD4$^+$CD25$^-$ T cells isolated from CD4$^+$ T cells in WT mice that had been treated with sublingual application of OVA protein. (**e**) Cell surface expression profile of KJ1-26$^+$ST2$^+$CD44$^+$ T cells among

$CD4^+$ T cells from *Rag2*$^{-/-}$*Foxp3*^{EGFP}DO11.10 mice that had been systemically immunized with or without OVA protein. (**f,g**) Cell dividing profile (**f**) and proportion (**g**) of eFluor™ 670-labelled KJ1-$26^+$$ST2^+$$CD44^+$ cells that had been cultured with or without $CD4^+$$CD25^+$ T cells or $CD4^+$$CD25^-$ T cells as prepared in Fig. 4i in the presence of OVA protein and cDCs for 3 days. (**h,i**) DEREG mice were treated with PBS or DT, and various tissues were obtained on the indicated days after the initial DT treatment. (**h**) Cell surface expression profile of $CD4^+$*Foxp3*$^{EGFP-}$ T cells among leukocytes in various tissues at the next day after each DT treatment. (**i**) Percentages of $CD4^+$*Foxp3*$^{EGFP-}$ T cells in various tissues in DEREG mice treated with DT compared with those in untreated DEREG mice. Data are obtained from five individual samples in a single experiment. $^*P < .05$ compared with untreated mice indicated as SLIT(-) or responder only. All data are representative of at least three independent experiments.

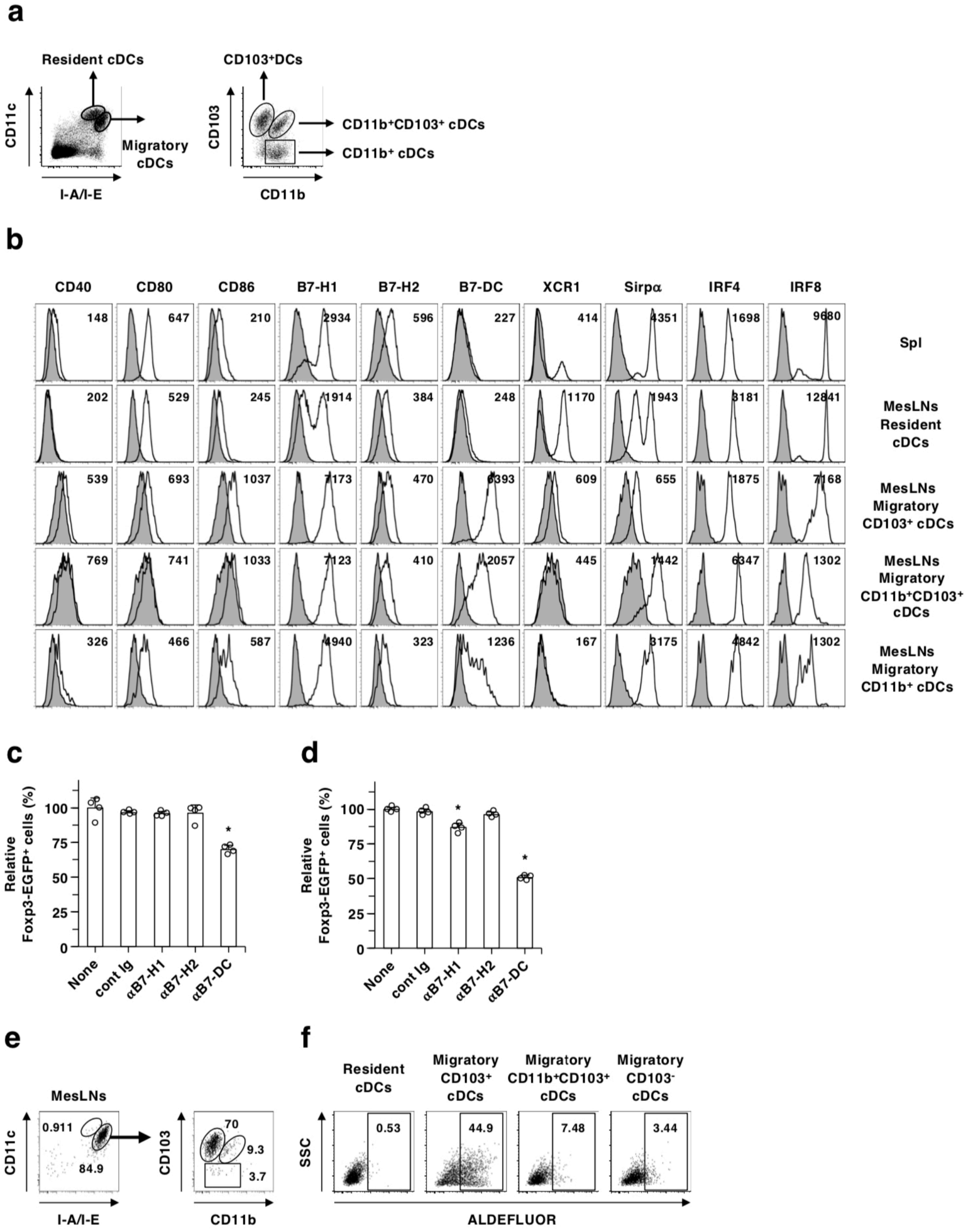

Supplementary Figure 8 Characterization of cDCs in MesLNs. (**a**) Cell surface expression profile of migratory I-A/I-E^{hi}CD11c^{med}CD11b^{+} cDCs, migratory I-A/I-E^{hi}CD11c^{med} CD11b^{+}CD103^{+} cDCs, migratory I-A/I-E^{hi}CD11c^{med}CD103^{+} cDCs, and resident I-A/I-E^{med}CD11c^{hi} cDCs in MesLNs. **(b)** Cell surface expression profile of cDCs in Spl and MesLNs in WT mice. **(c,d)** KJ1-26^{+}CD4^{+}*Foxp3*$^{EGFP-}$ T cells were cultured with ManLN (**c**) or MesLN cDCs (**d**) under pT$_{reg}$-polarized culture conditions for 5 days in the presence or absence of mAbs against B7 family members of costimulatory molecules. Proportion of KJ1-26^{+}CD4^{+}*Foxp3*$^{EGFP-}$ T cells. **(e,f)** Leukocytes in MesLNs obtained from WT mice were incubated with ALDEFLUOR for detection of ALDH activity. Cell surface expression profile of I-A/I-E^{hi}ALDEFLUOR^{+} cells (**e**) and ALDEFLUOR^{+} cells

in each cDCs subset in MesLNs (**f**). Data are obtained from five individual samples in a single experiment. *$P < .05$ compared among groups. All data are representative of at least three independent experiments.

Supplementary Table 1

Name	Supplier	clone	conjugate
B7-H1	BD	M1H5	PE
B7-H2	eBioscience	HK5.3	PE
B7-DC	eBioscience	TY25	PE
CD3ε	BD	145-2C11	FITC,PE,APC,BV421
CD4	BD	RM4-5	PE,PE-Cy7
CD11b	BD	M1/70	PE-Cy7,BV510
CD11c	BD	HL3	FITC,APC,BV421
CD19	Biolegend	6D5	Alexa-488,APC,BV510
CD25	BD	PC61	APC
CD40	BD	3/23	FITC
CD44	BD	1M7	BV510
CD45R/B220	BD	RA3-6B2	PE-Cy7,APC-Cy7
CD45.1	BD	A20	FITC,APC-Cy7
CD45.2	Biolegend	104	APC-Cy7
CD49b	BD	DX5	FITC,APC
CD64	Biolegend	X54-5/7.1	PE,APC
CD80	BD	16-10A1	FITC
CD86	BD	GL1	FITC
CD103	BD	M290	PE,APC,BV421
CXCR5	Biolegend	L138D7	APC-Cy7
DO-11.10 Clonotypic TCR	BD	KJ1-26	PE
FcεRIα	eBioscience	MAR-1	APC
GL7	Biolegend	GL7	Bacific Blue
IFN-γ	BD	XMG1.2	BV421
IgG1	BD	A85-1	APC
IL-4	BD	11B11	PE
IL-5	Biolegend	TRFK5	BV421
IL-13	BD	eBio13A	PE
IRF4	eBioscience	3E4	PE-Cy7
IRF8	eBioscience	V3GYWCH	PE-Cy7
I-A/I-E	BD	M5/114.15.2	APC-Cy7,BV510
Ly6G	Biolegend	1A8	FITC,PE-Cy7
PD-1	BD	J43	PE
PNA	MBL		Biotin
Siglec-F	Miltenyi	ES22-10D8	PE
Siglec-H	Biolegend	551	FITC,PE,Alexa-647
Sirpα	BD	P84	PE
ST2	BD	U29-93	APC,BV421
Streptavidin	BD		BV510
Vα2	BD	B20.1	PE
XCR1	Biolegend	ZET	FITC
