# Supplementary material for: Essential role of submandibular lymph node dendritic cells in protective sublingual immunotherapy against murine allergy
Source: Commun Biol. 2020 Dec 7;3:742. doi: 10.1038/s42003-020-01466-3 (PMC7721894; doi:10.1038/s42003-020-01466-3)
Supplement: Supplementary file 2 — Reporting Summary [file 42003_2020_1466_MOESM2_ESM.pdf]

nature research

Corresponding author(s): Katsuaki Sato

Last updated by author(s): Oct 7, 2020

Reporting Summary

Nature Research wishes to improve the reproducibility of the work that we publish. This form provides structure for consistency and transparency in reporting. For further information on Nature Research policies, see our Editorial Policies and the Editorial Policy Checklist.

Statistics

For all statistical analyses, confirm that the following items are present in the figure legend, table legend, main text, or Methods section.

n/a	Confirmed	
[ ]	[x]	The exact sample size (*n*) for each experimental group/condition, given as a discrete number and unit of measurement
[ ]	[x]	A statement on whether measurements were taken from distinct samples or whether the same sample was measured repeatedly
[ ]	[x]	The statistical test(s) used AND whether they are one- or two-sided<br>*Only common tests should be described solely by name; describe more complex techniques in the Methods section.*
[x]	[ ]	A description of all covariates tested
[ ]	[x]	A description of any assumptions or corrections, such as tests of normality and adjustment for multiple comparisons
[ ]	[x]	A full description of the statistical parameters including central tendency (e.g. means) or other basic estimates (e.g. regression coefficient) AND variation (e.g. standard deviation) or associated estimates of uncertainty (e.g. confidence intervals)
[x]	[ ]	For null hypothesis testing, the test statistic (e.g. *F*, *t*, *r*) with confidence intervals, effect sizes, degrees of freedom and *P* value noted<br>*Give P values as exact values whenever suitable.*
[x]	[ ]	For Bayesian analysis, information on the choice of priors and Markov chain Monte Carlo settings
[ ]	[x]	For hierarchical and complex designs, identification of the appropriate level for tests and full reporting of outcomes
[x]	[ ]	Estimates of effect sizes (e.g. Cohen's *d*, Pearson's *r*), indicating how they were calculated

Our web collection on statistics for biologists contains articles on many of the points above.

Software and code

Policy information about availability of computer code

Data collection	FACSuite software (ver1.0.6), FACSDiva software (ver8.0)
Data analysis	FlowJo 9.9.6 (Flow Cytometry analysis) and Microsoft Excel for Mac ver.15.37 (Graphs, statistics).

For manuscripts utilizing custom algorithms or software that are central to the research but not yet described in published literature, software must be made available to editors and reviewers. We strongly encourage code deposition in a community repository (e.g. GitHub). See the Nature Research guidelines for submitting code & software for further information.

Data

Policy information about availability of data

All manuscripts must include a data availability statement. This statement should provide the following information, where applicable:

- Accession codes, unique identifiers, or web links for publicly available datasets
- A list of figures that have associated raw data
- A description of any restrictions on data availability

Provide your data availability statement here.

Field-specific reporting

Life sciences study design

All studies must disclose on these points even when the disclosure is negative.

Sample size	A minimum of biological triplicates was used, the minimum needed to be able to perform statistics. For in vivo studies, the maximum number of animals available per treatment group were used to counteract biological variation.
Data exclusions	No data were excluded.
Replication	All attempts at replicating data were successful.
Randomization	For all studied C57BL/6 and BALB/c mice were purchased in batches of 10-20 mice and randomly distributed to cages.
Blinding	Blinding was not possible in this study as the experimenters treating the mice were the same as those analyzing the data. The treatment groups had to be clearly identified throughout the study to prevent cross contamination to be able to draw conclusions. However, statistics were performed in an unbiased manner.

Reporting for specific materials, systems and methods

We require information from authors about some types of materials, experimental systems and methods used in many studies. Here, indicate whether each material, system or method listed is relevant to your study. If you are not sure if a list item applies to your research, read the appropriate section before selecting a response.

Materials & experimental systems

n/a	Involved in the study	
[ ]	[x]	Antibodies
[x]	[ ]	Eukaryotic cell lines
[x]	[ ]	Palaeontology and archaeology
[ ]	[x]	Animals and other organisms
[x]	[ ]	Human research participants
[x]	[ ]	Clinical data
[x]	[ ]	Dual use research of concern

Methods

n/a	Involved in the study	
[x]	[ ]	ChIP-seq
[ ]	[x]	Flow cytometry
[x]	[ ]	MRI-based neuroimaging

Antibodies

Antibodies used	Fluorescent-dye-conjugated antibodies were purchased from BD (USA) (anti-B220, RA3-6B2, cat#557683, lot#22218; anti-B7-H1, M1H5, cat#558091, lot#82177; anti-CD3ε, 145-2C11, cat#562600, lot#8151862; anti-CD4, RM4-5, cat#553049, lot#69861, cat#552775, lot#8260833; anti-CD11b, M1/70, cat#552850, lot#8233702, cat#562950, lot#8038628; anti-CD11c, HL3, cat#550261, lot#04245, cat#562782, lot#8232678; anti-CD19, 1D3, cat#553786, lot#78439; anti-CD25, PC61, cat#557192, lot#42928; anti-CD40, 3/23, cat#553790, lot#15854; anti-CD44, 1M7, cat#563114, lot#8330958; anti-CD45.1, A20, cat#563775, lot#41071; anti-CD49b, DX5, cat#58295, lot#5089894; anti-CD80, 16-10A1, cat#553768, lot#04497; anti-CD86, GL1, cat#553691, lot#0000067499; anti-CD103, M290, cat#562771, lot#8213969; anti-DO-11.10 Clonotypic TCR, KJ1-26, cat#551772, lot#18954; anti-IgG1, A85-1, cat#560089, lot#5128838; anti-PD-1, J43, cat#551892, lot#0000063296; anti-Sirpα, P84, cat#560107, lot#02357; anti-ST2, U29-93, cat#566310, lot#8072829; anti-Vα2 TCR, B20.1 cat#553289, lot#78868; anti-IFN-g, XMG1.2 cat#563378, lot#6021864; anti-IL-4, 11B11, cat#554435, lot#0000060097; Streptavidin, cat#554060, lot#57973, cat#563261, lot#5156902), Biolegend (USA) (anti-B220, RA3-6B2, cat#103222, lot#B270362; anti-CD3ε, 145-2C11, cat#100306, lot#B215536, cat#100312, lot#B260199; anti-CD45.2, 104, cat#109852, lot#B247809; anti-CD64, X54-5/7.1, cat#139303, lot#B191539; anti-CXCR5, L138D7, cat#145505, lot#B168063; anti-GL-7, GL7, cat#144613, lot#B209951; anti-I-A/I-E, M5/114.15.2, cat#107627, lot#B248054; anti-Ly6G, 1A8, cat#127606, lot#B188856; anti-Siglec-H, 551, cat#129605, lot#B248184; anti-XCR1, ZET, cat#148209, lot#B231647; anti-IL-5, TRFK5, cat#504311, lot#B217463), eBioscience (USA) (anti-B7-H2, HK5.3, cat#12-5985-81, lot#E014592; anti-B7-DC, TY25, cat#12-5986-82, lot#1946688; anti-CD3ε, 145-2C11, cat#61-0031-80, lot#E19379-101; anti-FcαRIα, MAR-1, cat#12-5898-83, lot#E01909-1460; anti-IL-13, eBio13A, cat#2-7133-81, lot#E02109-1632; anti-IRF4, 3E4, cat#25-9858-80, lot#1936923; anti-IRF8, V3GYWCH, cat#25-9852-80, lot#2068058), or Miltenyi (Germany) (anti-Siglec-F, ES22-10D8, cat#130-102-274, lot#5160713134).<br>Biotinylated lectin was purchased from MBL (Japan) (PNA, cat#J1001012, lot#1936923).<br>Cell proliferation dye was purchased from eBioscience (USA) (eFluorTM670, cat#65-0840-85, lot#1984395).
Validation	All antibodies are commercially available and have been validated by suppliers and previous publications.

Animals and other organisms

Policy information about studies involving animals; ARRIVE guidelines recommended for reporting animal research

Laboratory animals	The following 8- to 12-week-old female mice were used in this study; C57BL/6 and BALB/c mice, B6.FVB-Tgltgax-DTR/EGFP Lan/J (CD11c-DTR/EGFP) mice, B6.CD45.1+OT-II OVA-specific TCR transgenic mice, B6.CD45.1+Foxp3EGFPOT-II OVA-specific TCR transgenic mice (B6.CD45.1+Foxp3EGFPOT-II mice), Rag2-/-Foxp3EGFPDO11.10 OVA-specific TCR (KJ1-26 clonotype) transgenic BALB/c mice, and C57BL/6-Tg(Foxp3-DTR/EGFP)23.2Spar/Mmjax (DEREG) mice.
Wild animals	N/A
Field-collected samples	N/A
Ethics oversight	University of Miyazaki

Note that full information on the approval of the study protocol must also be provided in the manuscript.

Flow Cytometry

Plots

Confirm that:

- [ ] The axis labels state the marker and fluorochrome used (e.g. CD4-FITC).
- [ ] The axis scales are clearly visible. Include numbers along axes only for bottom left plot of group (a 'group' is an analysis of identical markers).
- [ ] All plots are contour plots with outliers or pseudocolor plots.
- [x] A numerical value for number of cells or percentage (with statistics) is provided.

Methodology

Sample preparation	To prepare single-cell suspensions, Spl, MLN, and lung were digested with 400 U/ml collagenase type III (Worthington Biochemical) at 37 °C for 20 or 40 min, respectively, and were ground between glass slides. Splenocytes were treated with RBC lysis buffer (Sigma-Aldrich) before suspension. For the isolation of leukocytes from siLP, and colonic LP, intestines were opened longitudinally, washed to remove fecal content, and cut into small pieces. To remove epithelial cells, intestinal segments were treated in PBS containing 10 % FCS, 20 mM HEPES, 100 U/ml penicillin, 100 μg/ml streptomycin, 1mM sodium pyruvate, 20 mM EDTA and 10 μg/ml polymyxin B (Calbiochem) with continuous stirring at 37 °C for 20 min in a water bath. After wash with PBS, remaining tissues ware incubated with 400 U/ml collagenase type III, 250 mU/ml dispase (Life Technologies), and 100 μg/ml DNase I (Roche Diagnostics) for 20-30 min at 37 °C in a water bath. The cell suspension was prepared by forcing through a 100-μm cell strainer, washed with PBS, resuspended in 10 ml of 30% Percoll (GE Healthcare) and overlaid on 2 ml of 70% percoll in a 15-ml tube. Percoll gradient separation was performed by centrifugation at 780 g for 20 min at room temperature. The LP leukocytes were collected at the interface of the Percoll gradient and washed with RPMI1640/10% FCS, and used immediately for experiments. Peripheral blood mononuclear cells (PBMCs) were collected from blood by the use of Lympholyte-M (Sedarlane). For the isolation of bronchoalveolar lavage (BAL) leukocytes, the trachea was cannulated and lavaged two times with 1 ml sterile cold PBS. For the isolation of oral macrophages, oral tissues were collected and treated with collagenase type III (400 U/ml), dispase (250 mU/ml; LifeTechnologies) and DNase I (100 μg/ml) at 37°C for 45 min. CD11c+ DCs were purified by AutoMACS with mouse CD11c (N418) Microbeads (Miltenyi Biotec). Subsequently, ManLNs CD11c+ DCs were sorted into migratory I-A/I-EhiCD11cmedCD11b+ cDCs, I-A/I-EhiCD11cmedCD103+ cDCs and resident I-A/I-EmedCD11chi cDCs with high purity (each >99%) using a FACSAriaII cell sorter (BD Biosciences) with fluorescein-conjugated mAbs (BD Biosciences). CD4+ T cells were purified from splenocytes of WT mice, B6.CD45.1+OT-II mice (CD45.1+Va2+OT-II CD4+ T cells), B6.CD45.1+Foxp3EGFPOT-II mice (CD45.1+Foxp3EGFPVa2+CD4+ T cells), and Rag2-/-Foxp3EGFPDO11.10 mice (KJ1-26+CD4+Foxp3EGFP- T cells) with mouse CD4 T lymphocyte Enrichment Set-DM and (BD Biosciences). In some experiments, CD4+ T cells were sorted into CD4+CD25+ T cells and CD4+CD25- T cells with high purity (each >99%) by a FACSAriaII cell sorter with fluorescein-conjugated mAbs (BD Biosciences). Similarly, CD45.1+Va2+CD4 +Foxp3EGFP- T cells (CD45.1+OT-II CD4+Foxp3EGFP- T cells) were purified from CD45.1+Foxp3EGFPVa2+CD4+ T cells with >99% purity by FACSAriaII cell sorter, respectively.
Instrument	FACSVerse (for analyzing) or FACSAria II (for sorting) from BD Bioscience were used for data collection.
Software	FACSuite software (ver1.0.6) or FACSDiva software (ver8.0) was used for data collection, and FlowJo (ver9.9.6) was used for data analysis.
Cell population abundance	For flow cytometry analysis of leukocytes, a minimum of 500 cells per final gate was acquired. Purity confirmed by FACS was >99% after sorting.
Gating strategy	All samples were pre-gated as singlet and live (PI negative) events. Further gates for lymphocytes are indicated in the axes or legends of each figure.

- [x] Tick this box to confirm that a figure exemplifying the gating strategy is provided in the Supplementary Information.
